# Supercritical Fluid Applications in the Design of Novel Antimicrobial Materials

**DOI:** 10.3390/molecules25112491

**Published:** 2020-05-27

**Authors:** Irena Zizovic

**Affiliations:** Faculty of Chemistry, Wroclaw University of Science and Technology, Wybrzeze Wyspianskiego 27, 50-370 Wroclaw, Poland; irena.zizovic@pwr.edu.pl

**Keywords:** supercritical fluid, carbon dioxide, antibacterial activity, bacterial resistance, multidrug resistance, antibacterial materials, material design, green chemistry

## Abstract

Bacterial resistance to antibiotics is one of the biggest problems in the modern world. The prevention of bacterial spreading from hospitals to the community and vice versa is an issue we have to deal with. This review presents a vast potential of contemporary high-pressure techniques in the design of materials with antimicrobial activity. Scientists from all over the world came up with ideas on how to exploit extraordinary properties of supercritical fluids in the production of advantageous materials in an environmentally friendly way. The review summarizes reported methods and results.

## 1. Introduction

The application of supercritical fluids is a powerful tool in the development of novel materials with antimicrobial activity desperately needed in the time of increasing bacterial resistance to antibiotics and the dramatic appearance and spread of not only multidrug-resistant (MDR) but also pandrug-resistant (PDR) bacterial strains. MDR is defined as the resistance to at least one antibiotic from at least three different categories, while PDR is defined as non-susceptibility to all drugs in all antimicrobial categories [1]. According to the World Health Organization (WHO), antibiotic resistance is one of the biggest threats to global health, food security, and development today and it can affect anyone, of any age, in any country [2]. As stated in the Centers for Disease Control and Prevention (CDC) Antimicrobial Resistance Threats Report for 2019 [3], more than 2.8 million antibiotic-resistant infections occur in the U.S. each year and more than 35,000 people die as a result. The report lists 18 antibiotic-resistant bacteria and fungi into three categories based on the level of concern to human health—urgent, serious, and concerning. Common to all urgent threats is that nearly all those infections happen in patients who recently received care in a healthcare facility, identifying hospitals as places where MDR strains occur and from which they spread to the community. According to the CDC, the main endangered categories are patients who have surgery (among them, 1.2 million women who had a caesarean section in 2017), chronic conditions (e.g., diabetes), organ transplant recipients, patients who receive dialysis treatment, and people receiving chemotherapy [4]. As reported by the WHO regional office in Europe, the health burden of infections caused by antimicrobial resistance in the European Union is similar to that of influenza, tuberculosis, and HIV/AIDS combined. In 2015, there were 670,000 antibiotic-resistant infections in the European Union, which resulted in 33,000 deaths [5]. The problem of bacterial resistance to antibiotics is not related to humans and hospitals only. It is also an urgent issue in veterinary hospitals and clinics and, among others, the question of how to treat companion animals is raised.

This review aims to present high-pressure technologies involved in the design of unique antibacterial mats and the results obtained to encourage new ideas and projects toward an extensive application of these materials. It is important to stress that one of the techniques to be presented, Supercritical Solvent Impregnation (SSI), found its place on the industrial scale in wood impregnation [6,7,8,9] and textile dyeing [10,11,12]. The application of SSI in these industries transfers the production from the conventional, which generates enormous amounts of waste effluents into the modern fabrication of high-quality products with zero waste and with considerably lower energy demands. The extraordinary properties of supercritical fluids such as high density, near-zero surface tension, and high diffusivities enable the uniqueness and numerous advantages of the materials obtained. Especially carbon dioxide is of interest because of its favorable critical properties (31 °C and 7.38 MPa), availability, nontoxicity, and nonflammability. As it will be illustrated, due to its beneficial physicochemical and transport properties, supercritical carbon dioxide (scCO_2_) is used as a solvent, transport medium, polymer plasticizer, swelling agent, foaming agent, reaction medium, or antisolvent in an environmentally friendly way and without waste generation.

Impregnating solid matrices with active substances from liquid solutions by conventional techniques is associated with some significant drawbacks, such as using organic solvents, low penetration due to the surface tension of liquid phase, heterogeneous dispersion, and generation of high quantities of liquid and solid waste. Other conventional polymer impregnation techniques include the mixing of impregnates during polymeric synthesis or processing. Despite the simplicity, these methods have certain deficiencies, such as using organic solvents, which have to be removed to acceptable limits, undesired reactions, and degradation. ScCO_2_ applications can overcome most of the mentioned drawbacks in conventional processing. Besides the avoidance of organic solvents usage, deep penetration into the solid matrix, and zero waste generation, the additional advantages of these techniques are the possibility to work at relatively low temperatures, easy and complete separation of CO_2_ simply by the pressure reduction, no need for the drying step, as well as the possibility to tailor physical properties of many polymers.

The high-pressure technologies to be surveyed in this review may play a significant role in the prevention of emergence and spreading of MDR and PDR strains in public health objects, including hospitals as well as veterinary hospitals, by innovative antimicrobial mats design. There is a broad spectrum of potential applications for the novel materials, from wound dressings, medicinal textiles, frequently touched surfaces, air-conditioning filters, and surfaces for different usage, to medical devices like catheters, which may prevent the occurrence of infections in both human and animal patients, etc. From the technology point of view, any active substance or a combination may be used in the material design. According to the properties of the selected substance(s) and foreseen application, an appropriate base-material is to be chosen. The development of high-pressure processing then depends on both (e.g., hydrophilic or hydrophobic active principle, polymer behavior under high pressure, etc.), as it will be exemplified in further text. From the microbiology point of view, a single bioactive substance or a combination of active principles can be considered as well. Here, it is worth mentioning that, with the emergence of MDR bacterial strains, natural components and plant extracts have come into the focus of scientific interest again, after being “forgotten” in the antibiotic era [13,14,15,16]. Namely, the plant extracts are of particular clinical value nowadays because they generally do not confer resistance [15]. It was also reported that a combination of plant extracts and ineffective antibiotics might have an outcome in antibacterial activity against resistant strains [13,14].

Thus, as it will be presented, active substances used may be small molecules, natural extracts, antibiotics, nanoparticles, antibacterial polymers, antibacterial dyes, or other chemicals synthesized in conventional processes or in scCO_2_ as a reaction medium. Thymol and its isomer carvacrol were used in a considerable number of scientific studies as active substances for the design of antibacterial mats due to several reasons. These substances were reported to have significant antibacterial activity against both Gram-positive and Gram-negative bacteria [17,18,19] and are generally recognized as safe (GRAS status) by the Food and Drug Administration (FDA) [19].

## 2. Supercritical Solvent Impregnation (SSI)

**Figure 1:**
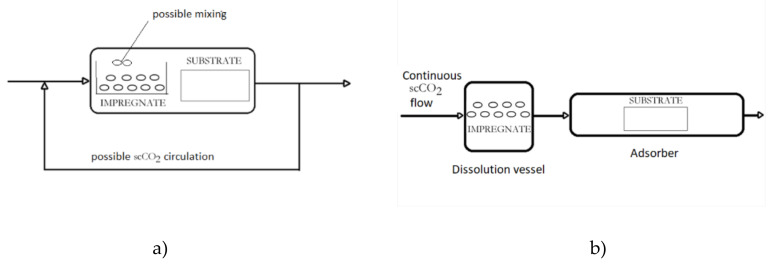
Simplified presentation of Supercritical Solvent Impregnation (SSI) modes: (**a**) batch; (**b**) semi-continuous.

This technique provides broad possibilities for material design when active substances are soluble in scCO_2_. In this process, the active substance is dissolved in scCO_2_ and the supercritical solution is brought to contact with a solid phase to be impregnated. The process may be conducted in a batch or semi-continuous mode (Figure 1). The supercritical fluid easily penetrates the solid phase due to the absence of surface tension, carrying the active component into the matrix. If there is a possibility of hydrogen bonding between the active substance and the solid (e.g., polymer chains), high loadings of the active substance may be achieved [20]. At the same time, the solid phase can be impregnated through the whole volume, which is a significant advantage of SSI over the conventional impregnation techniques where surface tension prevents liquid penetration into the solid matrix. If there is no possibility of hydrogen bonding, the active substance can be deposited in the solid phase simply by the decompression. Decompression and CO_2_ transfer from the supercritical into the gas phase lead to the decrease of the solubility of the active component in CO_2_ and its precipitation in the solid phase. Besides, no liquid effluent is generated in this process and no drying step is needed, which makes SSI environmentally friendly with considerably reduced energy requirements in comparison to the conventional impregnation processes. Thanks to these advantages, breakthroughs were made in the wood industry (Superwood, Hampen, Denmark) in spruce wood treatment as well as in the textile industry (DyeCoo, Weesp, Netherlands) in the dyeing of all kind of synthetic fabrics and yarn by applying this technology on the industrial scale.

### 2.1. Impregnation of Textiles and Fibers

It is difficult to control bacterial spreading in hospitals, particularly if we keep in mind that most of the Gram-positive bacteria can persist around one month on dry surfaces and that Gram-negative bacteria persist somewhat longer while fungal pathogens may survive up to 4 months [21]. Taking into account that textiles are also suitable substrates for bacterial and fungal growth, the usage of medicinal textiles is strongly recommended to protect patients.

SSI has been shown as feasible in textile impregnation with antimicrobial agents. Milovanovic et al. reported the considerable antibacterial activity of cotton gauze modified with thymol [22] and carvacrol [23] in a batch process. The gauze loaded with 11%wt. of thymol was obtained after 2 h of impregnation at 35 °C and 15.5 MPa. Longer impregnation time of 24 h led to the gauze loading with 19.6% of thymol. Despite the high thymol loadings, SEM micrographs revealed no visible crystals on the cotton fibers, indicating thymol distribution on the molecular level. Due to the establishment of hydrogen bonding between the hydroxyl group of thymol and cotton, high loadings of fibers were obtainable. Both samples (11% and 19.6% of thymol) showed strong antimicrobial action against tested strains of *Escherichia coli*, *Staphylococcus aureus*, *Bacillus subtilis*, *Enterococcus faecalis,* and *Candida albicans* [22]. In the case of carvacrol, higher pressures were needed to obtain high loadings. After 24 h of impregnation at 50 °C and 30 MPa, cotton gauze with 14.4% of carvacrol was obtained. The samples tested were shown to be effective against *E. coli* and *S. aureus* [23].

Polypropylene (PP) nonwoven materials are widely used in medical practice like in disposable surgical gowns, shoe covers, facemasks, drapes, head covers, etc. [24]. Markovic et al. reported a method to produce PP nonwoven medicinal textile with antibacterial activity and also excellent wettability [25]. Chemically inert nature of PP fibers was altered by corona discharge at atmospheric pressure resulting in the introduction of polar groups on the fiber surface and hydrophilicity increase. Corona-treated PP and a control (PP) were impregnated with thymol in a batch process with scCO_2_ at 35 °C and 15.5 MPa during 4 h. Surprisingly, there was no difference in the impregnation kinetics between the samples, and thymol loadings up to 11% were obtainable. However, crystals of thymol were spotted on the fiber surface using SEM images, indicating lower loadings in the fiber itself. This result is in accordance with the chemical properties of polypropylene and the absence of the possibility for hydrogen bonding with thymol. Still, samples with around 7% of thymol for both PP and corona pretreated PP nonwoven fabrics provided maximum microbial reduction (99.9%) against *E. coli*, *S. aureus,* and *C. albicans*. The wetting time of PP nonwoven material exceeded 20 min, whereas the corona modified PP was wetted immediately [25].

Markovic et al. [26] reported results on the impregnation of polyamide nanofibers with thymol in supercritical and liquid carbon dioxide. High-pressure impregnation provided superior thymol loadings of the electrospun nanofibrous mats (up to ∼60%) compared to the conventional immersion methods (∼2%). Experiments were performed with scCO_2_ at a temperature of 35 °C and pressures of 10 and 20 MPa as well as with liquid CO_2_ at 25 °C and 7 MPa. Operating time was varied in the range from 0.5 h to 4 h. The results indicated fast nanofiber impregnation, whereby already after 30 min of the SSI at pressures of 7, 10, and 20 MPa, thymol loadings reached the values of 6.5%, 23%, and 32%, respectively. The higher the pressure, the higher the rate of impregnation and the higher the loading. By tentative selection of operating conditions, it was possible to fabricate polyamide nanofibers with desired thymol loading ranging from 6% (at 7 MPa and 25 °C) to 60% (at 20 MPa and 35 °C). When conditions of 20 MPa and 35 °C were applied to the SSI of the conventional polyamide fabric during 0.5 h, the thymol loading of only 1.05% ± 0.24% was reached. This was remarkably lower compared to the thymol loading in nanofibers (around 32%), indicating a great advantage in the application of electrospun nanofibrous materials to the design of functionalized materials. The samples with loadings higher than 20% provided strong antibacterial activity against *E. coli*, *S. aureus*, and *C. albicans* [26].

As previously mentioned, plant extracts have been recognized as an important source of antibacterial agents. Sanchez-Sanchez et al. [27] reported results on the SSI of polyester textiles with mango leaf extract previously obtained by supercritical fluid extraction (SFE) with CO_2_ in the presence of methanol as a cosolvent. The impregnation process was conducted in a batch mode in the presence of methanol as well. The influence of pressure, temperature, and decompression on the chemical profile of loaded components and biological activity of the fabric was investigated. The highest activity against *E coli.* demonstrated material impregnated at 50 MPa and 55 °C for 22 h. This sample was characterized by the highest loading of total polyphenols and the highest contents of mangiferin and iriflophenone [27].

**Figure 2:**
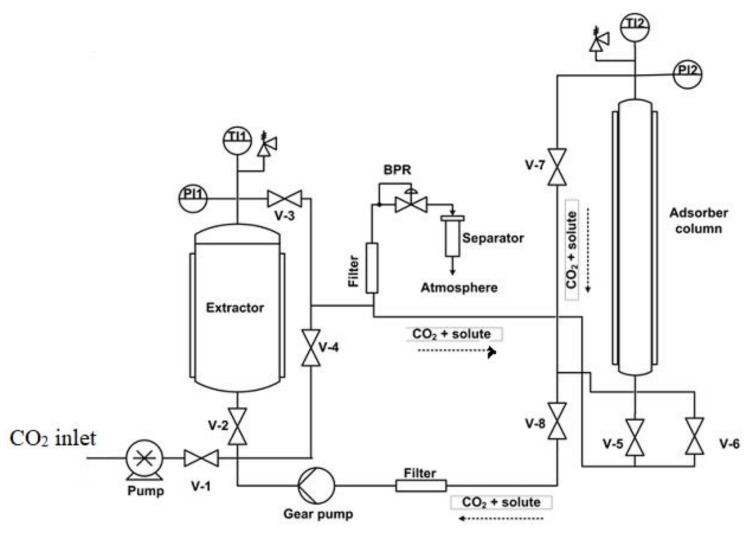
Integrated supercritical fluid extraction - impregnation (SFE-SSI) process.

Since plant extracts are of great importance as the source of bioactive components, as previously mentioned, Fanovic et al. [28] developed a process for integrated extraction and impregnation using scCO_2_ (SFE-SSI). The strategy of the process design was based on minimizing the loss of the extract in the tubes, vessels, and exchangers of the equipment by directly using the scCO_2_-extract solution leaving the extractor vessel for the impregnation. The coupling of SFE and SSI enables avoidance of the intermediate decompression step in the separate SFE and SSI processes (decompression after SFE) and consequently leads to energy and time savings. One of the process schemes is presented in Figure 1 (HPEA 500 unit, Eurotechnica GmbH). The extractor is filled with the raw material from which a target substance is to be extracted, and the adsorption column is filled with a solid to be impregnated by the extract from the prior extraction step. In the experimental setup shown in Figure 2, a backpressure regulator (BPR) is placed after the adsorption column, and a gear pump is provided for circulation of supercritical solution through both vessels or optionally through the adsorption column only. In this setup, the extractor and adsorber operate at the same pressure while the contact time may differ. The flow of the supercritical solution may be maintained through both vessels or the adsorber only. Since extraction is usually a much faster process than adsorption, such an option is often of interest. In the case that different process conditions are required in the extractor and adsorber, a BPR can be placed between the vessels. The integrated process has been successfully applied to add antibacterial properties to a variety of polymeric and textile materials [28,29,30,31,32,33,34]. Cotton gauze and polypropylene nonwoven fabric were impregnated with thyme extract rich in thymol, whereby the extract loadings of 9% and 5% were obtained for cotton and polypropylene, respectively [29]. Hop extract, characterized by strong antibacterial properties [35], was successfully incorporated into the polypropylene fibers with the loadings around 8% [31] using the combined process as well.

Gittard et al. [36] developed a method for antifungal textiles fabrication using silver deposition in scCO_2_. Silver precursors Ag(hepta) and Ag(cod)(hfac) soluble in scCO_2_ were investigated. The SSI of cotton fabrics with precursors, in a batch mode, was performed at 21 MPa and 40 °C, with the impregnation time ranging from 10 to 15 h. After the SSI followed by the system decompression, the impregnated precursor reduction was performed with a mixture of scCO_2_ and hydrogen. SEM imaging revealed both a thin layer of silver over the entire fabric surface and a scattering of silver aggregates on regions of the fabric surface. The product obtained was effective against *C. albicans*. The authors concluded that scCO_2_ processing might be used to impart antifungal functionality to textiles used in wound dressings. Also, hospital uniforms containing antifungal textiles might be used to prevent the spread of fungal infections in hospitals, nursing homes, and other healthcare settings [36].

A considerable and significant contribution to the development of antibacterial fibers by SSI was given by Chen et al. [37,38,39,40] and their work on the synthesis of antibacterial polymer molecules soluble in scCO_2_ and posterior SSI of different fibers with these polymers. The advantage of fibers coated with antibacterial polymers in comparison to the ones impregnated with smaller molecules is in the durability and allowance for a larger number of washing cycles. This is of importance for textiles like linen and uniforms in hospitals, especially if we bear in mind that textiles are suitable substrates for bacterial and fungal growth under the appropriate moisture and temperature conditions [41]. Antibacterial polymers can confer durable coatings through long-chain entanglements with surface molecules of materials. Substrates swell considerably but do not dissolve in scCO_2_, which is favorable to the impregnation of antibacterial polymers [40].

Polysiloxanes are soluble in scCO_2_ [42,43] and also widely employed in the textile industry due to their high gas permeability, low toxicity, and excellent stability. In the study [37], the antibacterial precursor was first synthesized from poly(methylhydrosiloxane) in a hydrosilylation reaction with *tert*-butyl acrylate. The ester groups of the precursor were hydrolyzed into carboxyl groups for conjugating with tert-butylamine molecules via amide bonds. Upon chlorine treatment with *tert*-butyl hypochlorite, the N−H bonds of the amides were transformed into biocidal N−Cl groups to generate the *N*-halamine polysiloxane. The impregnation of cotton fibers with *N*-halamine polysiloxane was performed in a batch mode at 25 MPa and 50 °C for 3 h. The experimental setup provided stirring of *N*-halamine polysiloxane during the process. The results showed *N*-halamine coating was about 60 nm thick, exhibited excellent stability and durability toward washing cycles, and the rechargeability of lost active chlorines was good. The antibacterial layer provided potent biocidal activities against *S. aureus* and *E. coli*. The study proved the feasibility of the SSI in cotton coating with the antibacterial polymer. It highlighted the SSI advantages over the conventional chemical treatments, which employed covalent bonding of antimicrobial groups to surfaces that generated contamination due to the use of toxic solvents and occasional decomposition of materials [37].

In another study [38], an *N*-halamine precursor 3-(3-hydroxypropyl)-5,5-dimethylhydantoin was synthesized and reacted with poly(methylhydrosiloxane) to produce a biocidal polysiloxane with 5,5-dimethylhydantoin-based *N*-halamine pendants. The polymer was coated onto polyethylene fibers in a batch SSI process at 28 MPa and 50 °C overnight. The thickness of the coating layer of about 73 nm allowed for effective biocidal activities against *S aureus* and *E. coli*. The coat on the polyethylene substrate was stable toward washing cycles, storage, and UV irradiation, and the rechargeability of lost active chlorines was good [38].

Further, Chen et al. synthesized polysiloxane with quaternized *N*-halamine moieties [39] and 4-ethyl-4-(hydroxymethyl)oxazolidin-2-one-based *N*-halamine polysiloxane [40] for the antibacterial coating of polypropylene via SSI. In both studies, the impregnation process was performed at 28 MPa and 50 °C overnight, whereby the mixing of the antibacterial polymer was provided. The coatings enabled effective biocidal activities against *S aureus* and *E. coli*. Both studies identified SSI as applicable to the modification of inert PP fibers without the need for pretreatments and providing durable interfacial properties of the fibers.

Another approach to the addition of antibacterial properties to fibers is in the application of antibacterial dyes. Elmaaty et al. [44] reported results on the preparation of antibacterial hydrazono propanenitrile dyes and their usage in the SSI of polyester fabrics. The fabric was impregnated in a batch SSI process, whereby the optimum conditions were found to be the temperature of 120 °C and pressure of 15 MPa with the impregnation time in the range 1–3 h. Depending on the dye, the SSI was performed with pure CO_2_ or with the addition of methanol as a cosolvent. Excellent results were obtained related to the color quality as well as the antibacterial activity against *S. aureus* and *E. coli* [44]. In the following study, Elmaaty et al. [45] investigated antimicrobial disperse dyeing of polyamide (Nylon 6) textiles in supercritical and liquid CO_2_. The process was conducted at pressures 5, 10, and 15 MPa; temperatures of 80, 100, and 120 °C, for time 1–3 h, and with hydrozono propanenitrile dyes concentration of 2–6% over the weight fiber. The best results were obtained at higher temperatures and pressures. In comparison to conventional dyeing from water solution, the scCO_2_ dyeing was shown to be superior related to the quality of the product obtained (color strength) and the quantity of spent dye. Besides, there was no salt and dispersing agent adding in the scCO_2_ process as well as no waste generation. Obtained fabrics exhibited antibacterial activity against *E. coli*, *S. aureus*, *P. aeruginosa*, and *B. subtilis* [45]. In the next study [46], Ma et al. presented viable dyeing of ultra-high-molecular-weight polyethylene (UHMWPE) fabric in scCO_2_ at a pressure of 20 MPa and temperature of 120 °C for 1–3 h. Five hydrozono propanenitrile dyes having antibacterial activity were applied. The color strength increased with the treating time as well as with the addition of decalin as a cosolvent. The fibers obtained showed antibacterial activity against *E. coli*, *S. aureus*, and *B. cereus* [46].

### 2.2. Impregnation of Polymeric Forms Other than Textiles and Fibers

In this segment, the review will start from cellulose acetate as an illustrative example of the versatility in material fabrication offered by SSI. In the case of thymol and carvacrol as antibacterial agents, this versatility is based on a wide range of loadings that could be obtained due to the possibility of hydrogen bonding between the polymer and active substance. Carbonyl and hydroxyl groups of cellulose acetate provide hydrogen bonding between polymer chains (intermolecular bonding). However, the hydroxyl group of thymol or carvacrol may establish hydrogen bonding with the functional groups of cellulose acetate as well, providing the possibility of high polymer loadings with these substances. Higher loadings cause a larger number of newly established hydrogen bonds with the active substance, while at the same time, intermolecular bonding between polymer chains becomes weaker. This phenomenon leads to polymer swelling. Milovanovic et al. [20] showed that maximal cellulose acetate loading with thymol was around 72%wt. The impregnation was performed in a batch mode at 35 °C and pressures of 10 and 20 MPa. The higher the pressure, the faster the impregnation, but maximal loading was the same and independent of pressure. The polymer swelling started with thymol loadings around 9%, agglutination of polymer beads began around 55%, and the soft-melt-like state occurred with 60% of thymol. After the decompression, the sample with 72% of thymol was in a solid state but swollen and changed shape. SEM imaging revealed the disappearance of the polymer porous structure with high thymol loadings caused by the increased mobility of the polymer chains. Thanks to this wide range of possible loadings, a variety of materials for different purposes may be fabricated. Thymol release studies indicated the release time from one to 21 days in water [20] and up to three days in the simulated gastric and intestinal fluids (hydrochloric acid and phosphate buffer saline) [47] depending on the loading. The impregnated samples showed antibacterial activity against 23 tested bacterial strains, including Methicillin-resistant *S. aureus* (MRSA), which causes severe infections in humans and animals [20,47], as well as *C. albicans* [20].

Similar results were obtained with cellulose acetate and carvacrol [48]. The SSI was performed in a batch mode at 50 °C and pressures of 10, 21, and 30 MPa. The rate of impregnation increased with the pressure increase. However, maximal carvacrol loading (around 60%) was not affected by the pressure applied. The samples showed considerable antibacterial effect against Gram-positive and Gram-negative bacterial strains, including MRSA. Samples containing around 30% of carvacrol showed excellent antibacterial activity while preserving a porous structure with submicron pore diameters [48].

As cellulose acetate loaded with thymol was indicated as a promising antibacterial material, Zizovic et al. [49] continued research towards the fabrication of thymol loaded polymeric films capable of preventing biofilm formation. Such polymers could be posted, for instance, to frequently touched places in hospitals. Biofilms are aggregates of microorganisms embedded in a self-produced extracellular polymeric substances (EPS) matrix that are adherent to each other and/or biotic or abiotic surface [49,50]. In a biofilm, bacteria can efficiently evade the immune system and can be up to 1000-fold more resistant to antibiotics and disinfectants than planktonic (free-living) cells [51]. The main components of the EPS matrix are water (97%), polysaccharides, phospholipids, several proteins, and extracellular DNA [52]. Bacteria capable of producing biofilms mostly cause chronic infections, which are characterized by persistent inflammation and extensive tissue destruction [49]. In the study [49], the focus was on *Pseudomonas aeruginosa* and MDR *S. aureus* recognized as pathogenic bacteria of critical and high priority, respectively [53]. Cellulose acetate films were produced using the solvent-casting method. The challenge was to create a film that could be loaded with a sufficient quantity of thymol to prevent biofilm formation, but at the same time, to keep its shape, bearing in mind that cellulose acetate undergoes swelling and softening with high thymol loadings. The results showed that it was possible to produce thymol loaded cellulose acetate films with anti-biofilm properties against *P. aeruginosa* and *S. aureus*. The film selected, with 30% of thymol and loaded by the SSI at 15.5 MPa and 35 °C, showed excellent anti-biofilm activity, ensuring inhibition of bacterial attachment to the film’s surface for all tested strains. The study included MRSA isolates that were previously categorized as excellent biofilm-producers and were resistant (besides to β-lactam antibiotics) to metronidazole and clindamycin and a clinical isolate of *P. aeruginosa* DM50 with a high ability to form biofilm and resistant to metronidazole, clindamycin, and amoxicillin. The study showed the feasibility of the SSI and solvent-casting method in film preparation. It is also important to stress that such high loadings of thymol in polymeric films, required to provide desired antibacterial action, are possible to obtain only via SSI. By thymol addition to the casting solution, loadings of only a few percent are attainable.

The next step in the design of antibacterial films or tapes might be their production by extrusion. As active compounds, both thymol and carvacrol can be used. The advantage of carvacrol usage is in its liquid state under the atmospheric pressure. In the following study [54], the cellulose acetate film with the best results in the SSI thymol [49] was successfully loaded with carvacrol as well. Loadings higher than 30% were obtained at 21 MPa and 50 °C. It was shown that the rate of decompression played an essential role in the process. The slower the decompression, the higher the loading. This relation was postulated for systems where a hydrogen bonding between the active substance and polymer was possible. In the opposite case, faster decompression usually means higher loading [54,55].

**Figure 3:**
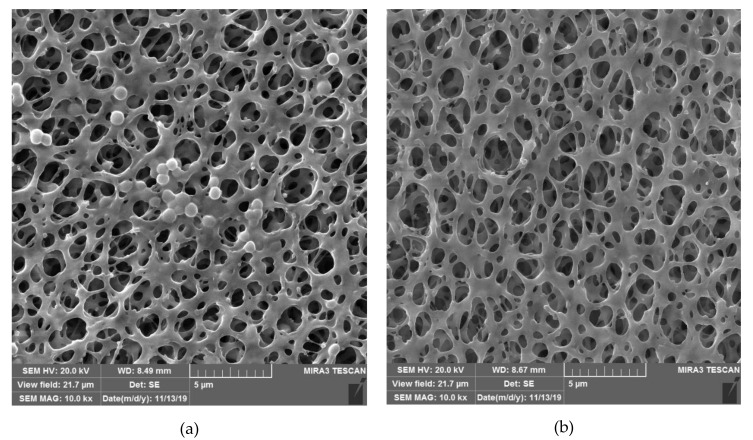
SEM images of the neat cellulose acetate-based polymer (**a**), and the cellulose acetate-based polymer loaded with thymol (**b**), after the exposure to *S. aureus* [56].

To illustrate the potential of materials obtained by SSI in the prevention of biofilm formation, SEM micrographs of a cellulose acetate-based polymer are presented in Figure 3 [56]. In Figure 3a, the neat polymer (control) after the exposure to *S. aureus* is shown. The attachment of the bacteria to the porous polymer structure is visible. In the case of the polymer loaded with thymol (Figure 3b), no bacteria could be found in the matrix. Unlike the conventional impregnation with liquids, SSI offers deep penetration of the active component into the polymer matrix and its interaction with polymer functional groups, leading to high loadings of finely distributed active component (on the molecular level) and high efficiency.

A recent study of Darpentigny et al. [57] revealed the potential of cellulose nanofibril porous materials as thymol carriers. Four types of nanocellulose materials (nanopapers, cryogel from water suspension, cryogel from tert-butyl alcohol suspension, and aerogel) were synthesized, characterized, and impregnated with thymol in a batch SSI process at 10 MPa and 40 °C for one hour. Aerogel obtained by supercritical drying at 10 MPa and 45 °C was characterized by the largest specific surface (160 m^2^/g) followed by the cryogel from tert-butyl alcohol suspension (97 m^2^/g). These structures showed the highest potential for thymol loading with values of 8.3% and 6.0%, respectively. Antimicrobial activity of both materials was proven against Gram-negative bacteria (*E. coli*), Gram-positive bacteria (*Staphylococcus epidermidis*), and a yeast (*C. albicans*). The results confirmed the feasibility of scCO_2_ application to the design (aerogel) and impregnation of nanocellulose 3D structures with bioactive molecules and might present an interesting solution for the design of active medical devices such as wound dressings [57].

Terzić et al. [58] applied scCO_2_ technology to the preparation of functional pH-sensitive chitosan-itaconic acid-methacrylic acid (Ch/IA/MAA) aerogels characterized with micron-size pores and their impregnation with thymol. Ch/IA/MAA hydrogels were obtained first, transferred to alcogels, and then dried in air to obtain xerogel or in scCO_2_ to obtain aerogels. The scCO_2_ drying consisting of 10 min of static and 120 min of dynamic drying at 11 MPa and 45 °C followed by the decompression at a rate of 1 MPa/min resulted in an advantageous aerogel with favorable swelling kinetics and elasticity, compared to the xerogel and aerogels obtained at other decompression rates and drying times. The aerogel was subsequently loaded with thymol (up to 4.6 wt.%) in a batch SSI process at 10 MPa and 35 °C with the impregnation time of up 5 h. In vitro studies of swelling in phosphate buffered saline (PBS) at 37 °C indicated a considerable potential of the obtained stimuli-responsive gel for topical administration of thymol [58].

Dias et al. [59] presented results on the development of natural-based wound dressings impregnated with thymol and quercetin as bioactive compounds using SSI. Film- and foam-like structures of N-carboxybutylchitosan and agarose were prepared and impregnated with supercritical (20 MPa, 40 °C) and near-critical (10 MPa, 30 °C) carbon dioxide for 3 h. To obtain polymers with both active compounds, thymol and quercetin were loaded simultaneously in the presence of ethanol as a cosolvent for quercetin. The supercritical conditions were indicated as favorable, while the foam-like N-carboxybutylchitosan structure was characterized with the highest loading capacity. The quantities of quercetin loaded simultaneously with thymol and in the separate process were similar (around 27 µg/mg polymer), while the quantity of thymol loaded in the simultaneous process was slightly lower (22 µg/mg polymer) than in a separate process (25 µg/mg polymer). This is a consequence of the presence of ethanol, which may have increased thymol solubility in scCO_2_ and lowered the partition coefficient between the polymer and supercritical phase. The study revealed the feasibility of simultaneous SSI for the selected system [59].

Tsutsumi et al. [60] reported results on the synthesis of biodegradable copolymer Poly(L-lactide-ran-cyclic carbonate) and its impregnation with *d*-limonene as an antibacterial agent. Outstanding controlled release materials were developed with statistical random copolymers of L-lactide (L-LA) with cyclic carbonate (CC) (2,2-dimethyltrimethylene carbonate (2,2-DTMC) or tetramethylene carbonate (TEMC)). The impregnation was performed in a batch mode at 40 °C and 20 MPa for 3 h. The loadings obtained were up to 5.3% and were the highest in copolymers with L-LA content of around 80% [60].

Poly(lactic acid) (PLA) as a biodegradable and thermoplastic polymer received considerable attention in SSI applications and especially in the design of added value films since it has excellent processability, a reasonably good barrier, and mechanical properties for a broad spectrum of applications [61]. Polymeric films with antibacterial properties are of considerable interest for the storage and packaging of sterile items in hospitals. Yu et al. [62] applied SSI to prepare roxithromycin-loaded biodegradable PLA films. The effects of impregnation time, operating temperature, and pressure on the drug loading were investigated. Optimal conditions for the incorporation of this antibacterial drug into PLA films were 30 MPa, 343 K, and impregnation time of 2 h, whereby the maximal loading was approximately 10.5%. The SSI process was implied to be a promising technique for the preparation of drug-loaded biodegradable polymer surfaces and matrices for antibacterial therapeutic implants [62].

Torres et al. [63] investigated the SSI of PLA films with thymol. The impregnations were performed at pressures of 9 and 12 MPa; the temperature of 40 °C; and decompression rates of 0.1, 1.0, and 10 MPa/min for 3 h. Depending on the impregnation conditions, thymol was incorporated into the films at loadings from 13.5% to 20.5%, whereby the highest loadings were obtained with the slowest decompression rate. The impregnation of thymol in PLA using scCO_2_ was indicated as a promising technique to prepare active biodegradable materials for a wide range of applications [63]. Villegas et al. [64] applied the same SSI condition range [63] to incorporate cinnamaldehyde into PLA films. Depending on the process parameters, the loadings obtained were from 8% to 13% *w*/*w*. Higher pressure and slower decompression rate resulted in higher loadings. The incorporation of cinnamaldehyde improved the thermal, structural, and mechanical properties of the PLA films. The tested samples showed strong antibacterial activity against *E. coli* and *S. aureus* [64]. In the next study, Villegas et al. [65] reported results on the SSI of bionanocomposite films based on PLA reinforced with nanoclay C30B (5.0% *w*/*w*) with thymol and cinnamaldehyde. The SSI was performed at 12 MPa, 40 °C, and the decompression rate of 1 MPa/min for 3 h. Loadings obtained were around 11% for cinnamaldehyde and 17% for thymol. The samples showed strong antibacterial activity against *E. coli* and *S. aureus.* All bionanocomposites were fully disintegrated in compost, showing their possible application as compostable active films [65].

Milovanovic et al. [66] reported results on the preparation of poly(lactic acid)/poly(ε-caprolactone) (PLA/PCL)-blended films and their impregnation with thymol and in a batch SSI process as well as with thyme (*Thymus vulgaris*) extract in an integrated SFE-SSI process. The batch impregnation experiments were performed at 10 MPa, 40 °C with films of various PCL contents (0, 1, 5, and 10 wt%) and with different operating times to maximize thymol loading and to retain a compact structure and good thermal stability of the films. The PCL content of 5% and the impregnation time of 5 h provided the highest thymol loading of 35.8% while retaining good thermal stability at temperatures up to ~150 °C. The film displayed strong bactericidal properties against *B.subtilis* and *E. coli*. In the experiments performed in the integrated SFE-SSI process, the impregnation time, PCL content, and scCO_2_ flow regime were varied to maximize the thyme extract loading. However, the obtained loading of around 5% was not sufficient to inhibit the growth of the abovementioned bacterial strains [66].

Fanovic et al. [28] presented results on the development of PCL scaffold with antibacterial activity by an integrated SFE-SSI process. In this, so-called “3 in 1” process, 3 processes were integrated: SFE of an extract with strong antibacterial activity from lichen *Usnea lethariiformis*, PCL impregnation with the extract, and formation of the desired porous structure of the impregnated polymer by the proper decompression rate (controlled polymer foaming). The SFE and the impact of decompression rate on the pore size distribution were investigated before the integrated process design. The optimal process parameters of 30 MPa and 40 °C for the SFE and of 15 MPa and 35 °C with the decompression rate of 0.5 MPa/min for the SSI resulted in impregnated scaffolds with the average pore diameter of around 340 µm. The optimal flow regime was found to be continuous scCO_2_ flow for 2 h with posterior scCO_2_ recycling through the system for 1 h. The extract loadings of around 2.8% slowed down the multiplication of tested MRSA strains [28]. In the next study, Fanovic et al. [34] reported the application of an integrated SFE-SSI process to the fabrication of microporous polycaprolactone–hydroxyapatite (PCL–HA) scaffolds with antibacterial activity. The HA content and particle size as well as the operating conditions of the integrated process were optimized regarding the amount of the impregnated antibacterial agent (*Usnea lethariiformis* extract) and antibacterial activity against selected MRSA strains. The optimal processing parameters were found to be the SFE (30 MPa/40 °C) and adsorption (17 MPa/35 °C) with continuous scCO_2_ flow through both vessels for 2 h, followed by 1 h of the scCO_2_ recycling through the system at 17 MPa and 35 °C. The scaffold obtained under these conditions had the extract loading of 5.9% and the best bactericidal effect on the tested MRSA strains [34].

Another polymer shown to be suitable for SSI with bioactive substances, especially in the form of film, is low-density polyethylene (LDPE). Torres et al. [67] investigated the impregnation of linear LDPE (LLDPE) with thymol in supercritical and near-critical carbon dioxide. Impregnations were performed in a batch process at 40 °C and pressures from 7 to 12 MPa for 4 h. The thymol loadings obtained were in the range from around 1.5% in liquid CO_2_ (at 7 MPa) to 3.8% in scCO_2_ (at 12 MPa) [67]. Goni et al. [68] reported results on the SSI of LLDPE with eugenol as an antimicrobial agent and antioxidant. The SSI was performed in a batch mode at 45 °C and pressures of 10, 12, and 15 MPa for 4 h. The decompression rates were 0.5, 1, and 5 MPa/min. The loadings obtained varied in the range 1–6%, with higher values at lower decompression rate and higher pressure [68]. In another study, Medeiros et al. [69] investigated the incorporation of eugenol rich clove bud (*Syzygium aromaticum*) essential oil in LLDPE by SSI. The batch process was performed at temperatures of 25, 35, and 45 °C and pressures of 15 and 25 MPa for 4 h. The essential oil was present in the quantity of 2% and 10% of the CO_2_ mass. The highest oil loading (around 4%) was obtained at 45 °C and 15 MPa, with 10% of clove essential oil in the system [69].

Rojas et al. [70] reported results on the SSI of LDPE nanocomposites with different concentrations of organo-modified montmorillonite (OM-MMT) (2.5 and 5.0% (*w*/*w*)) with thymol in a batch process. The SSI was performed at 12 MPa and 40 °C for 1 h. Two decompression rates were evaluated (10.0 and 1.0 MPa/min). The achieved loadings were in the range of 0.36–1.19%. The highest loading of thymol (1.19%) was obtained employing the lower decompression rate for the sample with 5% of the OM-MMT nanoclay. It was observed that the OM-MMT nanoclay content was the main factor that affected the amount of incorporated thymol [70]. In the subsequent study [71], the influence of pressure and impregnation time on the thymol loadings in LDPE nanocomposites were investigated. The SSI was performed in a batch mode at 40 °C and pressures of 9, 12, and 15 MPa, with the impregnation times ranging from 0.5 to 5 h and with two different decompression rates (10.0 and 1.0 MPa/min). The optimal process parameters resulting in the highest loading (around 1.62%) were 12 MPa, 5 h, and 1.0 MPa/min. The incorporated quantity of thymol was sufficient to provide antibacterial activity against tested *S. aureus* and *E. coli* [71].

Bierhalz et al. [72] investigated possibilities to incorporate natamycin into alginate films. Three different techniques were considered: the conventional loading method (natamycin was added directly to the polymeric aqueous film-forming solution), the immersion procedure, and SSI with and without the addition of cosolvent (ethanol, 10% mol). The SSI was performed in a batch mode at 20 MPa and 40 °C for 2.5, 4, and 14 h. The decompression rate was 0.5 MPa/min. The conventional method led to films heterogeneities with high surface roughness, and the immersion technique had several disadvantages, including low drug loadings. The SSI for 14 h in the presence of ethanol provided desired loadings (around 1.6%) and led to homogeneous films, visually attractive and translucent [72]. Similar superiority of the SSI technique over conventional technologies was reported by de Souza et al. [73] for the incorporation of cinnamaldehyde into cassava starch biocomposite films. The SSI was performed in a batch mode at 35 °C, 15 and 25 MPa, for 3 h, and with two decompression rates (0.1 and 1 MPa/min). The highest loading (around 0.25%) was obtained at the higher pressure and faster decompression rate [73]. SSI of a starch material with thymol was the topic of the study reported by Milovanovic et al. [74]. Corn and tapioca starch hydrogels prepared at different temperatures were converted to the acetogels and subsequently dried with scCO_2_ or air to obtain aero- or xerogels, respectively. The gels were impregnated with thymol in a batch SSI at 15.5 MPa and 35 °C for 24 h. The loadings obtained were in the range of 1.15–4.02% [74]. In the subsequent study [33], curry plant (*Helichrysum italicum*) extract, known for its antibacterial properties, was incorporated into the starch xerogel in the integrated SFE-SSI process. Both processes, the extraction and impregnation, were performed at 35 MPa and 40 °C. The highest loading of around 1.26% was obtained for the scCO_2_ circulation through the extractor and adsorber during 5 h and with the plant material/starch mass ratio of 10 [33]. Varona et al. [55] impregnated starch modified with the n-octenyl succinate (OSA) in powder form with lavandin essential oil. The effects of pressure (10–12 MPa), temperature (40–50 °C), and lavandin oil to starch mass ratio (0.2–1) were studied. The loadings obtained were in the range from 25 to 150 mg lavandin oil/g OSA-starch [55].

With the same success as in the SSI of fibers [37,38,39,40], Chen et al. [75] applied SSI to the coating polyethylene terephthalate (PET) with biocidal quaternary ammonium/N-chloramine polysiloxane. The polymer soluble in scCO_2_, a polysiloxane with both quaternary ammonium and N-chloramine, was synthesized in a three-step route and used for PET impregnation. The SSI experiments were performed in a batch mode at 28 MPa and 50 °C overnight and resulted in a 70-nm biocidal layer. The incorporation of quaternary ammonium and N-chloramine provided a synergetic biocidal performance of the coated material allowing for strong antibacterial activity against *S. aureus* and *E. coli* in a short time. The polysiloxane interpenetration layer was stable, and the rechargeability of lost chlorine was good when the layer was subjected to repeated washing, storage, and UV irradiation [75]. Here, it is important to highlight that the modification procedure reported is environmentally friendly and applicable to other substrates since it does not rely on chemical linkage but on the interpenetration of the antibacterial polymer into the surface layers of the substrate swollen by scCO_2_.

The aim of the study reported by Xu et al. [76] was to chemically attach antibacterial agents to hydroxyl groups of cellulose, hemicellulose, and lignin to avoid leaching of antibacterial agents which inevitably occurs after physical adsorption in wood treatment. Nine quaternary ammonium compounds (QACs) containing at least one hydroxyl group were synthesized, and two of them with very strong antibacterial activity against *E. coli* were chemically attached to hemlock by using hexamethylene diisocyanate (HDI) as a linker via a carbamate/urethane linkage. ScCO_2_ was employed as the reaction medium and to facilitate penetration of the reactants (QACs and HDI) into the softwood cell walls. The reactions were performed at 100 °C and 41.4 MPa for 20 h using SSI, first for the linker and then for the QAC attachment. With the QACs being coated on the cell walls, the chemically modified wood demonstrated outstanding antibacterial activity, dimensional stability, and improved surface properties [76]. The study provided a unique method for the production of added value wood. Demonstrated bactericidal activity of the modified wood is of interest for utilization in hospitals as places of intensive bacterial multiplication and spreading.

## 3. Supercritical Assisted Impregnation (SAI) and High-Pressure Assisted Impregnation (HPAI)

**Figure 4:**
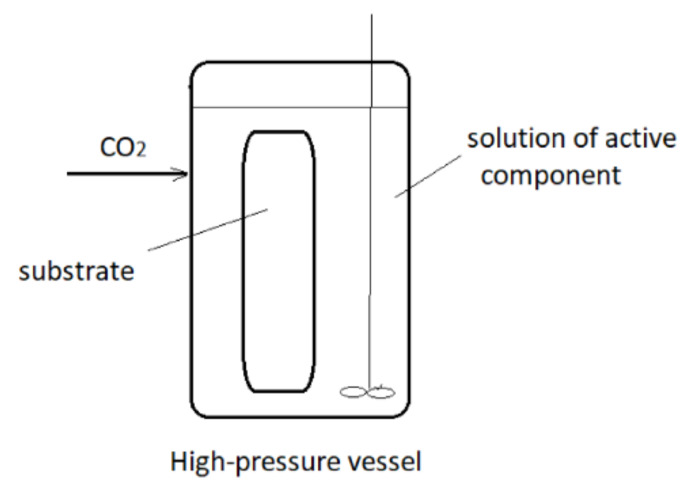
Simplified presentation of Supercritical Assisted Impregnation (SAI)/High-Pressure Assisted Impregnation (HPAI) process.

These techniques may be applied to the impregnation of active substances, which are less soluble in scCO_2_ or not soluble at all. In those processes, an active component is dissolved in an appropriate liquid solvent, and the liquid phase is brought to contact with a solid to be impregnated in the presence of supercritical or high-pressure carbon dioxide (hpCO_2_—CO_2_ under high pressure but not in the supercritical region). A simplified presentation of the process is presented in Figure 4. In this way, good transport properties of carbon dioxide in a liquid or supercritical state promote the contact between the liquid and solid. Quite often, swelling of the solid surface occurs, which also promotes the impregnation process. In the process, a considerably smaller quantity of the liquid phase to dissolve the active component is usually employed in comparison to the conventional impregnation from liquids. Also, a cosolvent may be added to scCO_2_ to enhance the interaction between the active substance and the supercritical phase. The main difference between the SSI and SAI techniques is the following: in SAI, a contact between 3 phases exists (solid substrate, supercritical phase, and liquid phase) no matter what is the solubility of the active component in scCO_2_. The active principle may be dispersible or soluble in scCO_2_. Unlike, in the case of SSI, the substrate is in contact with the supercritical phase only.

The first and essential application of HPAI was in leather tanning [77]. Leather is produced when an impregnate (tanning agent—usually chromium-III-salt) reacts chemically with collagens in pretreated animal hides in an aqueous solution. Leather manufacturing conventional process is exceptionally intensive concerning the consumption of resources, and an estimated overall amount of about 14 million m^3^ of wastewater per year is generated worldwide [77]. In the new process, the skins are contacted with a tanning solution and subsequently contacted with hpCO_2_ (>3 MPa) in rotating tanning drums. CO_2_ is partly diffusing into the skin and in the tanning solution. The leather of high quality is obtained already after 2 h of contact with CO_2_. There is no wastewater generation in the new process. In comparison to conventional method, consumption of the tanning agent is decreased for more than 50% and there is no need for the addition of sodium salt [77]. Further in text, results on the implementation of HPAI and SAI in the production of novel antibacterial materials will be presented.

Mölders et al. [78] applied carbon dioxide in a liquid (12 MPa, 20 °C) and supercritical state (12 MPa, 40 and 80 °C) to impregnate polycarbonate with silver nitrate as an antibacterial agent. The experiments were performed in a batch mode in a high-pressure view cell but also scaled up in a high-pressure vessel of 2 L. The samples were submerged in an ethanol solution of silver nitrate, heated, pressurized, and impregnated for 10 min. In parallel, submerging tests were performed under atmospheric pressure. Impregnation assisted by scCO_2_ was superior in comparison to the impregnation in liquid CO_2_ and far more superior than submerging at ambient pressure, providing silver content of around 23.4 mg/kg polymer. HPAI with liquid CO_2_ provided silver content of 2.4 mg/kg polymer while submerging under atmospheric pressure and 80 °C resulted in a content of 0.2 mg/kg polymer. The samples impregnated by both supercritical and liquid carbon dioxide showed strong antimicrobial activity against *E. coli*. Abrasion as well as UV-radiation and led to a loss of antimicrobial activity of the samples impregnated at 20 °C. However, the samples impregnated at 80 °C resisted the tests. The leaching of the samples was analyzed to determine the toxicity on humans, and the toxicity could not be confirmed [78]. These excellent results opened a way towards production of antibacterial surfaces which could be applied to many elements in hospitals such as doorknobs, switches, handrails, buttons, surfaces for placement of medical devices, etc.

The subsequent studies deal with the development of a novel class of antibacterial mats based on carbon nanomaterials and silver nanoparticles (NPs) [79,80]. Carbon nanotubes and nanofibers wrapped by silver NPs were fabricated with the assistance of scCO_2_ [79]. The SAI process was performed with the ethanol solution of the carbon materials, the silver precursor (AgNO_3_), and glucose as a reducer at 12 MPa and 65 °C for 3 h. The TEM and SEM images revealed that carbon nanotubes/AgNPs hybrids possess a preferable assembled structure. Experimental results demonstrated considerable antibacterial activity of tested materials against *E. coli* [79]. In the following study, Haldorai et al. [80] reported results on graphene oxide treatment with silver NPs in the presence of scCO_2_ to produce a material with photocatalytic and antibacterial activity. Graphene oxide was treated in an ethanol solution with AgNO_3_ and glucose as a reducer at 12 MPa and 65 °C for 3 h. The graphene oxide modified with silver NPs displayed an excellent visible-light photocatalytic performance in degrading Rhodamine 123 dye and acetaldehyde as well as significant antibacterial activity against *E. coli*, *S. aureus*, and *Listonella anguillarum* [80].

Based on the available literature survey, results on the SAI and HPAI applications are scarce but impressive. These techniques are a powerful tool yet to be applied to the design of novel materials. In the next section, the review will present combined processes of SAI/SSI and polymerization in scCO_2_, which opened possibilities for the design of unique antibacterial mats.

## 4. Supercritical Solvent Impregnation or Supercritical Assisted Impregnation Coupled with Polymerization in scCO_2_

The subsequent studies deal with the application of composite polymers known as interpenetrating polymer network (IPN) [81] in biomedical purposes. Solvent-free IPNs can be produced using scCO_2_ [81]. In this process, one or more monomers are dissolved or dispersed in supercritical or near-critical carbon dioxide and brought to contact with a polymer to be impregnated (SSI or SAI). The polymerization and crosslinking of monomers can be performed by a radical starter that can be impregnated into the polymer matrix simultaneously with the monomer(s). The polymerization reaction may be triggered by the temperature increase in the supercritical conditions upon the impregnation, consequently leading to the formation of IPN. Because there is no chemical bonding between the polymer and the network (between two polymers), each material retains its individual properties in the blend. This allows for a variety of applications for the novel type of materials synthesized in an environmentally friendly way [81]. The different behavior of the polymers in IPN in combination with the solvent-free appearance of the final product makes these materials especially attractive for the design of medical devices.

Steffenson et al. [82,83] demonstrated that silicone elastomers used in catheter production could be modified to form an IPN material with a poly(2-hydroxyethyl methacrylate) (PHEMA)-based hydrogel. Extruded silicone [82] and poly(dimethylsiloxane) (PDMS) silicone elastomer [83] were impregnated with (2-hydroxyethyl) methacrylate (HEMA) and ethylene glycol dimethacrylate (EGDMA) in the presence of cosolvent(s) and a radical starter in scCO_2_. The impregnation was performed at 40 °C and pressures 20–25 MPa for a time from 20 min to 16 h, depending on the contact between phases. The polymerization proceeded at 75 °C and 30–36 MPa for 3 h. Fabricated IPN materials retained mechanical properties similar to those of the original silicone elastomer while acquired the ability of the hydrogel to swell in aqueous media. PHEMA content was in the range of 13–38% (*w*/*w*). It was shown that the hydrogel formed an interconnected hydrogel network in aqueous media when the content of PHEMA was at least 25%. The optimized IPN material was loaded with the antibiotic ciprofloxacin, and the resulting drug release inhibited bacterial growth of *S. aureus* when placed on agar [82]. In the further study [83], it was demonstrated that samples containing 25% (*w*/*w*) hydrogel loaded in a 5 mg/mL ciprofloxacin medium inhibited *S. aureus* growth upon incubation in broth with high efficacy for 29 days whereby no biofilm was observed on the material. These substantially significant results opened a possibility for the design of novel medical devices for long-term clinical use.

In the next study, Stenger et al. [84] produced IPN catheters by the polymerization and crosslinking of PHEMA in silicone elastomer in scCO_2_ as previously described [82,83]. The system was loaded with dicloxacillin alone or in combination with thioridazine and tested against methicillin-sensitive *S. aureus* and MRSA. The drug-loaded IPN material was proven to be effective in in vitro experiments. Moreover, the IPN catheters were tested in a novel porcine model of central venous catheter-related infection, in which they were found to decrease the frequency of infection significantly [84].

The results presented on the preparation and application of IPN materials with controlled release of antibiotics are of the utmost importance bearing in mind that bacterial colonization with subsequent biofilm formation constitutes a severe and frequent problem associated with the use of many polymer materials commonly applied for medical devices [83]. Urinary tract infections are the most frequently occurring nosocomial infections [85]. During the long-term use of catheters, the risk of urinary tract infections increases rapidly over time and reaches 50% after 7–10 days [83,86]. To illustrate the significance, in the USA, approximately 250,000 of vascular catheter-related bloodstream infections occur annually associated with a mean hospital length stay of 22 days, increasing the hospital cost from US$ 3000 to 56,000 per patient, and with mortality rates of 12–25% for critically ill patients [83,87,88].

The use of carbon dioxide as a polymerization reaction medium has been investigated continuously since it is a green solvent with many advantages over conventional solvents [89,90]. Correia et al. [89] reported a method to obtain biocompatible 2-oxazoline-based oligomers quaternized with different amines using scCO_2_ as a reaction medium. Oligo(2-methyl-2-oxazoline) and oligo(2-bisoxazoline) quaternized with N,N-dimethyldodecylamine were shown to be very efficient biocidal agents showing fast killing rates against *S. aureus* and *E. coli*. In a further study, Correia et al. [91] presented a novel approach to the design of antibacterial materials by combining plasma technology, SSI, and polymerization in scCO_2_. In this study [91], oligo(2-methyl-2-oxazoline) quaternized with N,N-dimethyldodecylamine was grafted to a chitosan (CHT) scaffold. Chitosan scaffolds were prepared with the freeze-drying method, and subsequently, their surface was activated by argon plasma treatment. Upon the activation, the scaffolds were subjected to the SSI with the monomer 2-isopropenyl-2-oxazoline for 24 h at 18 MPa and 40 °C. After this grafting step, another monomer, together with an initiator, was introduced into the system, and the polymerization took place at 18 MPa and 65 °C for 20 h. In the final step, a tertiary amine was added to the reactor and the reaction was performed at 18 MPa and 40 °C for 20 h. The material obtained efficiently killed *S. aureus* and *E. coli* cells upon direct contact and prevented bacterial adhesion to the materials surface and biofilm formation. The material was shown to be suitable for water purification over ten cycles of reuse, efficient within minutes of contact and without leaching to the water [91].

Cationic antimicrobial peptides are promising antibacterial agents [89] and, as presented, can be synthesized and grafted to solid carriers in scCO_2_ [91]. Their mechanism of action is based on electrostatic forces and subsequent interaction between the cationic peptide and the anionic lipopolysaccharide in outer membrane of Gram-negative bacteria or the negatively charged teichoic acids attached to the thick layer of peptidoglycan present in the surface of Gram-positive bacteria [89,92]. It is believed that bacteria cannot develop resistance to these antibacterial polymers because the mechanism of action depends on the fundamental characteristics of the microbial cytoplasmic membrane. Therefore, the development of resistance would require bacteria to change their membrane structure completely [89,93].

## 5. Supercritical Foaming

Dissolution of scCO_2_ in polymers may increase chain mobility and induce polymer swelling in amorphous and semi-crystalline polymers, at the same time decreasing their melting point under the supercritical conditions [94]. Optionally, a cellular structure of the polymer matrix (foam) can be formed by inducing phase separation with a pressure and/or temperature change. Supercritical foaming was previously mentioned in connection to SSI of PCL [28]. In this part, it will be commented more since it is not connected with the SSI technique only, as it will be seen from the next example.

García-González et al. [95] reported results on the preparation of PCL-chitosan scaffolds containing vancomycin as an antimicrobial agent by scCO_2_ foaming, aimed for bone regeneration purposes. The foaming was performed from solid dispersions of PCL, chitosan, and the antibiotic. Powdered mixtures with different PCL, vancomycin, and chitosan contents were introduced into cylindrical Teflon molds, compacted, and exposed to scCO_2_ at 40 °C and 14 MPa for 1 h with subsequent decompression under the CO_2_ flow rate of 1.8 g/min. The obtained scaffolds showed a suitable combination of morphological (porosity, pore size distribution, and interconnectivity), and vancomycin release behavior, as well as the biological properties (cell viability and proliferation, osteo differentiation, and tissue-scaffold integration). The scaffolds sustained vancomycin release in PBS for more than two weeks and showed considerable antibacterial activity against *S. aureus* and *E. coli* [95]. This study exemplifies a method for the incorporation of a substance poorly soluble in scCO_2_ (vancomycin) into polymeric foams. Subsequent studies relate to the incorporation of a scCO_2_ soluble substance into polymer matrix by foaming and SSI as a one-step process.

Ivanovic et al. [94] reported results on the impregnation and foaming of PCL and polycaprolactone-hydroxyapatite (PCL-HA) composites with thymol in scCO_2_ for obtaining functional porous scaffolds. The effect of scCO_2_ sorption kinetics on the swelling, foam morphology, and thermal behavior of the PCL and PCL-HA materials was studied, whereby sorption isotherms were determined using a magnetic suspension balance at 10–30 MPa and 35–40 °C and thermal properties using high-pressure differential calorimetry (HP-DSC) at pressures 4.6–17.0 MPa. In the next step, SSI of PCL and PCL-HA with thymol was performed simultaneously with the foaming to produce scaffolds with antimicrobial properties and controlled microstructure. The pressures in the range 13–17 MPa and 10% of HA were proven to be favorable for the creation of scaffolds with satisfying foam microstructure (mean pore size ∼200–300 µm), filler distribution, and thymol loadings (12–18%) [94].

Milovanovic et al. [96] prepared foams loaded with thymol in a one-step SSI-foaming process from amorphous, medical grade poly(D,L-lactic acid) (PLA), and poly(D,L-lactic-co-glycolic acid) (PLGA). The impregnation performed with different CO_2_ densities (273–815 kg/m3) and short processing times (2 and 4 h) enabled thymol loading of 0.92–6.62%. The process was optimized for each polymer to obtain stable microcellular foams upon the system decompression. The highest thymol loading (6.62%) was obtained for the copolymer PLGA, whereby the sample exhibited controlled thymol release within 72 h in media having pH values from 1.1 to 7.4 [96].

## 6. Supercritical Drying of Metal-Carrying Gels

Synthesis of metallic nanoparticles is of great importance for the application in catalysis, electronics, and optics and for the design of materials with antibacterial properties [97]. The preparation of metal colloids by the reduction is a simple reaction. Still, the control of particle size, shape, and dispersion stability requires careful control of the synthetic conditions because the process is sensitive to balances between nucleation and crystal growth [97,98]. One approach to facilitate both the synthesis control and immobilization is the use of porous materials as reaction medium, which might be a hydrogel [97]. The hydrogel can further be transformed into an alcogel by the solvent exchange and subsequently to an aerogel by supercritical drying resulting in a highly porous added value material for a wide range of applications [97,99,100]. Aerogels are characterized by the small bulk densities (0.017–0.021 g/cm^3^), low thermal conductivities, big surface area (200–800 m^2^/g), and proven capability for the controlled release of incorporated substances [99,101,102,103].

Cai et al. [97] synthesized silver, gold, and platinum nanoparticles in the cellulose hydrogel by hydrothermal reduction by the cellulose itself (for silver at 80 °C for 24 h) or by adding a reductant (for gold and platinum). To produce aerogels, the water of metal-cellulose hydrogels was exchanged to ethanol, and two-step batch drying in carbon dioxide was applied. First, the ethanol was replaced with liquid CO_2_ at 5.3 MPa and 4 °C for 6 h and then supercritical drying took place at 10 MPa and 40 °C for 0.5 h, with subsequent slow decompression. The aerogels obtained were characterized by the high transmittance, porosity, and surface area as well as good mechanical strength [97].

Raman et al. [99] reported results on the synthesis of calcium-alginate aerogels augmented with zinc and silver for potential application in wound healing. By the combination of high-pressure gelation (room temperature, 50 MPa, for 24 h) and supercritical drying with a continuous flow of scCO_2_ (at 50 °C and 12 MPa for 2 h and under 20 g/min CO_2_ flowrate), hybrid Ca–Zn particles as well as hybrid Ca–Zn–Ag aerogel monoliths and particles were produced. The metal ions were released into supernatants upon the aerogels swelling in aqueous solutions in the amounts needed for a wound dressing [99].

In the subsequent study [100], pectin-TiO_2_ nanocomposite aerogels were prepared via the sol-gel process, consecutive solvent exchange step, and supercritical drying. The drying was performed at a temperature and pressure in ranges 50–60 °C and 11–13 MPa, respectively, for 5 h and with the scCO_2_ flow rate of 0.2 kg/h. In the presence of TiO_2_ nanoparticles, mechanical, thermal, and antimicrobial properties (against *E. coli*) of pectin-based aerogels were improved in comparison to the control ones. Thus, the aerogels may provide antibacterial protection and, to some extent, thermal protection due to the low thermal conductivity and may have a potential application in packaging for sensitive items [100].

## 7. Other Methodologies Applied to the Development of Antibacterial Materials

In this part, more ideas for the utilization of the extraordinary properties of supercritical fluids in the design of antibacterial materials will be presented. In the recent study, Li et al. [104] presented results on the synthesis of the hybrid Cu_2_O/TiO_2_ nanocomposites with the enhanced photocatalytic antibacterial activity against *Acinetobacter baumannii*. *A. baumannii* is Gram-negative bacteria, widespread, and multidrug resistant often found in intensive care unit, where it causes intra-hospital infections including sepsis, urinary tract infections, ventilator-acquired pneumonia, and wound infections [104,105]. In this study [104], a stable combined p-n Cu_2_O/TiO_2_ heterojunction was prepared by a supercritical solvothermal process in ethanol. The supercritical solvothermal process is regarded as a powerful tool for the synthesis of heterojunction materials with considerable advantages over conventional methods. Compared to the physical mixture, aqueous reduction, photochemical, and hydrothermal routes, all accompanied with weak combination, nonuniform size distribution, and easy aggregation, the application of supercritical fluids can provide a stable combination between Cu_2_O and TiO_2_ with the uniform dispersion and small crystal size resulting in the large special surface areas with a mesoporous structure and the expended visible-light absorption [104]. It is believed that the high rate of crystal nucleation without the easy crystal growth is the consequence of the high temperature and pressure applied in the supercritical state [104,106,107]. Cu_2_O/TiO_2_ composites were synthesized in supercritical ethanol at 243 °C and 6.4 MPa. In this process, Cu(NO_3_)_2_·5H_2_O and *tetrabutyl* titanate were dissolved into absolute ethanol and kept at the operating conditions for 70 min to complete the synthesis. The bactericidal activity of the 5.0% Cu_2_O/TiO_2_ sample in the case of *A. baumannii* was 100% under the visible-light irradiation within 30 min. Moreover, the 5.0% Cu_2_O/TiO_2_ nanocomposite displayed the significant visible-light antibacterial activities (up to 100% mortality in 30 min) against other pathogenic bacteria including *P. aeruginosa*, *E. coli*, and *S. aureus*. Based on the experimental findings, it was presumed that the Cu_2_O/TiO_2_ composite first led to the leakage of K^+^ ion with the disrupted permeability of the cell membrane and then induced the formation of inorganic compounds from the cell decomposition. The composites were shown to be durable due to the stable p-n Cu_2_O/TiO_2_ heterojunction obtained under the supercritical conditions. The durability and photocatalytic antibacterial activity of the composites present significant potential for the application in disinfection [104].

Bhartia et al. [108] used scCO_2_ for grafting semiconductor surfaces with monolayers of alkylthiols. Hydrogen-terminated semiconductor surfaces were exposed to alkylthiols dissolved in scCO_2_ at 100 °C and 10 MPa for the chemical reaction and establishment of the strong and nonpolar Si–S surface bond. The deposited monolayer on oxide-free silicon was stable, dense, and able to passivate the surface for more than 50 days (10 times than the conventional methods) without any oxide formation in the ambient atmosphere. The material resisted cell proliferation on the surface for more than 15 days and, besides the application in electronics, is envisaged for biomedical and antimicrobial applications. The inert nature of CO_2_, as the ideal contamination-free isolated processing environment for grafting better-quality monolayers, allowed for the production of superhydrophobic and bio-resistant surfaces [108]. In this environmentally free process, drawbacks of conventional technologies are overcome and the product of better quality is obtained.

Katayama et al. [109] used scCO_2_ to induce large pleat-like wrinkles on the surface of cotton fibers as support for nanoparticles. The cotton was immersed into the water first, and the treatment with scCO_2_ followed. The process parameters were optimized to produce the appropriate wrinkles. The favorable conditions were found to be a temperature of 40 °C, the pressure of 20 MPa, the contact time of 60 min, and a fast decompression rate of 0.80 MPa/min^−1^. It is assumed that the wrinkles occur due to the different degasification rates from the inner and surface parts of the fiber during the fast decompression. The material obtained was proven to be a suitable support for TiO_2_ nanoparticles of average 35 nm in diameter without the presence of binders [109].

Cuadra et al. [110] used scCO_2_ as an antisolvent to prepare a new adduct of isonicotinamide with copper(II) propanoate, a ligand complex with strong fungicidal properties. The precipitation was performed by introducing an ethanol solution of the components through a 100-µm nozzle into scCO_2_ at 40 °C and 10 MPa at a flow rate of 1 mL/min. ScCO_2_ dissolves in the ethanol, consequently decreasing the ligand complex solubility and leading to the precipitation. Applying the supercritical antisolvent (SAS) technique, crystals 100-fold smaller than those obtained by slow evaporation were produced, indicating a considerable bioavailability enhancement [110].

Imbuluzqueta et al. [111] employed liquid CO_2_ (10 MPa, 25 °C) as an antisolvent to produce a novel bioactive hydrophobic gentamicin-filled carrier. In this process, gentamicin was ion-paired with the anionic surfactant Bis(2-ethylhexyl) sulfosuccinate sodium salt (AOT) to obtain a hydrophobic complex (GEN–AOT). The solution of GEN–AOT in acetone was sprayed through a hollow cone nozzle into the CO_2_, resulting in the precipitation due to the antisolvent effect and allowing for GEN-AOT micronization. In a further step, the encapsulation of the obtained complex in PLGA nanoparticles was performed by the emulsion solvent evaporation method. The procedure provided NPs with GEN–AOT encapsulation efficiency of 100% and sustained release of the drug over 10 weeks. It was also shown that neither ion pairing, supercritical fluid processing, nor encapsulation in polymeric NPs affected the bactericidal activity of gentamicin against *E. coli* [111].

Saelo et al. [112] applied Rapid Expansion of a Supercritical Solution into a Liquid Solvent (RESOLV) process to obtain caffeic acid phenethyl ester (CAPE) nanoparticles. The mixture of CAPE, ethanol, and scCO_2_ at 17.3 MPa and 50 °C was expanded through a nozzle at 80 °C into distilled water. The obtained CAPE NPs were incorporated into methylcellulose films in the process of film preparation by the solvent casting method. Films containing 0.5% of CAPE NPs exhibited antimicrobial properties against *P. aeruginosa*, *C. albicans*, and *Listeria monocytogenes* [112].

Varona et al. [113,114] applied high-pressure techniques PGSS (Particles from Gas Saturated Solutions) and PGSS-drying to encapsulate lavandin (*Lavandula hybrida*) essential oil known for its antibacterial and antiviral properties. Carrier materials investigated were soybean lecithin, n-octenyl succinic anhydride (OSA) modified starch, PCL, and polyethylene glycol (PEG). PGSS was applied to the oil encapsulation into PCL [113] and PEG [114]. In this process, the lavandin oil and polymer were filled together in a high-pressure cell and intensively mixed in the presence of scCO_2_ for 2 h (the polymer was in a molten state) to reach phase equilibrium. Then, the mixture was depressurized, and due to the rapid expansion through a nozzle to ambient pressure, small particles were formed. The driving force for particle formation is the strong cooling as a consequence of the Joule Thomson effect produced during the expansion. It results in the polymer solidification and a covering layer formation around the essential oil droplets. PGSS-drying was applied to the oil encapsulation into OSA modified starch and soybean lecithin [113,114]. In this process, an oil-in-water emulsion was prepared in which the essential oil constitutes the dispersed phase and OSA-starch/soybean lecithin acts as a surfactant. The emulsion saturated with CO_2_ was contacted with the scCO_2_ in a static mixer and subsequently expanded through a nozzle. The expansion facilitated the formation of extremely fine droplets which dried very fast, while the polymer solidified encapsulating the essential oil. The results showed an enhancement of the antibacterial activity of lavandin oil against *E.coli*, *S. aureus*, and *Bacillus cereus* by the encapsulation due to the protection and control release provided by the carrier [113]. PGSS processes may provide polymer particles of micron size, filled with an antimicrobial agent, and suitable for spraying onto different surfaces.

Recent studies [115,116,117,118] demonstrated the feasibility of scCO_2_ application in liposome production. Conventional methods of liposome production suffer from drawbacks, such as the difficulty of controlling particle size distribution, micrometric dimensions, low stability, and high solvent residue [117]. To overcome these deficiencies, Santo et al. [115] and Trucillo et al. [116] developed a continuous supercritical assisted process called SuperLip (Supercritical assisted Liposome formation), characterized with good control of particle size distribution, possibility to produce liposomes on a nanometric or micrometric level, liposome stability of over one year, and solvent residue in liposomes lower than FDA limits [116]. The SuperLip process was successfully applied to the production of liposomes with antimicrobial activity loaded with vancomycin [116], amoxicillin [117], ampicillin, and ofloxacin [118]. In this process, an ethanol solution of phospholipids is brought to contact with scCO_2_ at 10 MPa and 40 °C in a saturator vessel first. The expanded liquid ethanol-scCO_2_ mixture is subsequently introduced into a high-pressure formation vessel operating at the same temperature and pressure conditions as the saturator. An aqueous solution with an active substance is injected through a nozzle into the formation vessel as well. The atomized droplets of water solution are quickly captured by the phospholipids contained in the fluid phase, creating lamellae around the inner core containing the drug. This is the key step of the scCO_2_-assisted process and an inversion of the traditional liposome production [117]. These inverted micelles, falling in a water bulk formed at the bottom of the vessel, are covered by a second lipids layer, completing the double-layer structure. The results showed that it was possible to control particle size distribution at the nanometric level, with an encapsulation efficiency of the drug up to 84% [117].

In the following study, Trucillo et al. [119] applied two scCO2-assisted techniques to load alginate aerogels with liposomes containing amoxicillin. The SuperLip process was used first to obtain amoxicillin loaded liposomes. In the next step, liposomes were entrapped in alginate hydrogels. After the water replacement with ethanol, obtained alcogels were subjected to supercritical drying to obtain aerogels. The results demonstrated that ampicillin release time from these meta-carriers was about four days or twice its release time from liposomes alone [119].

The studies presented in this review are listed in Table 1, whereby the main process parameters were provided.

## 8. Discussion

The high-pressure techniques presented are novel in the domain of antimicrobial mats design. Most of the references cited (around 93%) date from the last decade, and the earliest one was published in 2008. This review highlights the potential of the high-pressure methodologies in the development of new materials characterized by enhanced properties such as high loadings, extended release, and even distribution of an active component, durability, stability, etc. The extraordinary properties of scCO_2_, namely favorable critical parameters and transport properties as well as near zero surface tension allow for the design of materials that cannot be produced by other methods. In addition, the techniques are environmentally friendly, with no waste generation, and therefore are preferable for the industrial application.

In Table 2, the references are connected with the techniques applied. As can be seen, most of the references relate to SSI, which has already been applied on the industrial scale in the wood and textile industries, as previously mentioned. In the last few years, a significant potential of SAI and HPAI has been shown, revealing the successful application of high-pressure CO_2_ in the impregnation of poorly soluble in scCO_2_ substances. Recent reports also opened the door to the development of unique materials by coupling SSI/SAI and chemical reactions in scCO_2_. All the cited processes employed scCO_2_ as a supercritical solvent, except for the solvothermal method in which supercritical ethanol was used due to the high temperature demand of this process. All the techniques were shown to be promising for the development of antimicrobial mats in the future.

SSI is the most applied in material design and is a successfully scaled-up technique. However, it is restricted to active substances soluble in scCO_2_. The addition of cosolvent might improve the solubility of some polar compounds in scCO_2_, yet it complicates the processing on a larger scale. One possible direction for future research related to SSI in antimicrobial mats design is incorporating several active substances into a solid carrier. This is due to the current microbiology trend of investigating the synergistic antimicrobial activity of several compounds in tackling the bacterial resistance problem [13,14,15,16]. From the technology point of view, the impregnation may be conducted simultaneously at the highest pressure (corresponding to optimal solubility of the least soluble compound from the group) with all active compounds present in the solution or a fractional SSI process might be developed. In this process, impregnation starts from the highest pressure and the least scCO_2_ soluble component, followed by the pressure reduction and the impregnation with the second (more soluble) component. The fractional impregnation may also be applied to the incorporation of a combination of a hydrophilic and a hydrophobic substance. In this process, SAI or HPAI is applied first to deliver the hydrophilic component into the solid matrix from its solution (e.g., ethanol solution) with the assistance of carbon dioxide. In the next step, the SSI is applied to deliver the hydrophobic component into the solid matrix, without an impact to the content of the firstly impregnated compound.

Another direction for further research is a combination of extrusion and SSI. Namely, scCO_2_ with the dissolved active substance might be used in the supercritical-assisted polymer extrusion process. An example of a suitable substance for this purpose, with a broad spectrum of antimicrobial activity, is carvacrol. Since it is in a liquid state under the atmospheric conditions, carvacrol is easy to use on a larger scale, and there is no clogging after the decompression.

Textile dyeing with antimicrobial dyes [44,45,46], proven to be feasible using SSI, is promising for industrial production since the scale-up for this technology is known. Presented antibacterial properties of the obtained products as well as the existing needs for the added-value medicinal textiles ensure their wide applications.

As shown in studies [37,38,39,40,75], SSI was very efficient in polymer coatings with scCO_2_ soluble polysiloxanes with *N*-halamine side groups, yielding in the product resistant to washing cycles and abrasion, with proven antibacterial activity. The scale-up of this technology is more challenging compared to fiber dyeing. Here, we have surface coating with big molecules and not the impregnation with small molecules which easily penetrates bulk material. However, the product is advanced medicinal textile or the coated polymer surface nowadays very needed. Therefore, this application is also a candidate for further study and industrial application.

ScCO_2-_assisted impregnation (SAI and HPAI) is a recent technique with great potential in material design. For sure, it will be broadly explored in the future. Unlike SSI, it also enables the incorporation of poorly in scCO_2_ soluble substances due to the excellent transport properties of supercritical and liquid carbon dioxide as well as their interaction with the solid matrix. The recent study on the production of polycarbonate antibacterial surfaces [78] demonstrated the feasibility and plausibility of the process. The scale-up of these processes includes the optimization of contact between the phases and minimization of the liquid phase quantity. Rotating tanning drums in leather production is an example of a successful scale-up of the HPAI process [120].

The results obtained in the production of IPN materials [82,83,84] for catheters and other medical devices with antibacterial properties via SSI/SAI and polymerization in scCO_2_ are fundamentally important and promising for the full application. No serious issues are expected in scaling up the SSI or SAI for the silicone-based materials as well as in the polymerization in scCO_2_ conditions triggered by the temperature increase. The SAI might be scaled up similarly to leather tanning [120].

2-oxazoline-based oligomers quaternized with different amines using scCO_2_ as a reaction medium were shown to be efficient antibacterial agents [89]. The synthesis and grafting of these antibacterials to the chitosan surface were successfully performed using plasma technology, SSI, and scCO_2_ as the reaction medium [91]. This process might be of particular interest in the future since it is believed that bacteria cannot develop the resistance against these cationic peptides because the mechanism of action depends on the fundamental characteristics of the microbial cytoplasmic membrane [93].

Among the presented processes, grafting semiconductor surfaces with monolayers of alkylthiols in scCO_2_ [108] and the production of superhydrophobic and bio-resistant surfaces are of interest for both electronics and biomedical applications. The product is obtained in an environmentally friendly way.

The review was written from a technological point of view. Another one might be written from microbiology and dealing with the methodologies for the determination of antimicrobial activity. It is hard and often impossible to compare the antimicrobial activities of materials reported in different studies due to the various methods and conditions of microbiological investigations. Firm joint research of engineers, material scientists, and chemists with microbiologists, pharmacists, and medical staff is needed to obtain all required for the successful application of new material.

This review aims to stress the existence of new and advanced technologies in antimicrobial materials design and the results obtained. The investment costs in the high-pressure equipment are higher in comparison to the investments in conventional processes. However, the quality and efficiency of the products are higher in high-pressure applications. Besides, those are environmentally friendly technologies with no waste generation and with quite often lower operating costs due to the lower energy demands. If we manage to reduce the number of infections in patients using catheters longer than seven days and if we lower the number of people being infected in health care institutions and consequently we lower transmissions of these infections to the community, the economic burden to the society described in the introduction section will be reduced as well. A strong collaboration of governments and health organizations with scientists is needed in this topic.

## Figures and Tables

**Table 1:** Tabular presentation of cited results.

Active Substance	Technique and Main Process Parameters	Solid Material	Loading (Result)	Microorganism	Reference
Thymol	SSI, 35 °C, 15.5 MPa, 1–24 h	Cotton fibers	1.74–19.6%	*E. coli, S. aureus, B. subtilis, E. faecalis, C. albicans*	[22]
Carvacrol	SSI, 50 °C, 10–30 MPa, 1–24 h	Cotton fibers	4–14.4%	*E. coli, S. aureus*	[23]
Thymol	SSI, 35 °C, 15.5 MPa, 4 h	Polypropylene fibers	0.5–11.2%	*E. coli, S. aureus, C. albicans*	[25]
Thymol	SSI, 35 °C, 10 and 20 MPa, 0.5–4 h Near-critical, 25 °C, 7 MPa, 0.5–4 h	Polyamide nanofibers	22.6–59.2% 6.51–33.8%	*E. coli, S. aureus, C. albicans*	[26]
Mango leaf extract	SSI, 35 and 55 °C, 40 and 50 MPa, 22 h Methanol cosolvent	Polyester fibers	1.1–2.8% polyphenols	*E. coli*	[27]
Thyme extract	SFE-SSI, 35 °C, 15 MPa, batch 5 h	Cotton fibers Cellulose acetate Polypropylene fibers PCL Chitosan	7.18% 1.44% 4.78% 9.04% 0.96%		[29]
*Usnea barbata* extract Curry plant Lemon balm	SFE-SSI, 40 °C, 30 MPa, batch 5 h	LDPE Polypropilene fibers Cotton fibers	3.05% 3.99% 2.24%		[30]
Hop extract	SFE-SSI, 35 °C, 15 MPa, batch 5 h SFE-SSI, 50 °C, 29 MPa, batch 5 h	PCL Polypropylene fibers Starch xerogel	6.04% 4.36% 2.58%		[31]
Thyme extract Thymol Thymol Thymol	SFE-SSI, 110 °C, 30 MPa, 2 h batch + 2 h flow SSI, 35–110 °C, 10 and 30 MPa, 2–4 h SSI, 35 °C, 7.5 MPa, 2 h SSI, 35 °C, 15 and 30 MPa, 2 h	PLA PLA PLGA Starch	1.2% 4.9–6.6% 3.0% 14.7–31.9%		[32]
Ag(hepta), Ag(cod)(hfac)	SSI, 40 °C, 21 MPa, 10–15 h Reduction in H_2_ + scCO_2_	Cotton fabric	Silver coating	*C. albicans*	[36]
*N*-halamine polysiloxane	SSI, 50 °C, 25 MPa, 3 h	Cotton fibers	60 nm coating	*E. coli, S. aureus*	[37]
*N*-halamine polysiloxane	SSI, 50 °C, 28 MPa, overnight	Polyethylene fibers	73 nm coating	*E. coli, S. aureus*	[38]
*N*-halamine polysiloxane	SSI, 50 °C, 28 MPa, overnight	Polypropylene fibers	Coating	*E. coli, S. aureus*	[39,40]
Hydrazono propanenitrile dyes	SSI, 120 °C, 15 MPa, 1–3 h Methanol cosolvent	Polyester fabric	Dyeing	*E. coli, S. aureus*	[44]
Hydrazono propanenitrile dyes	SSI, 80–120 °C, 5–15 MPa, 1–3 h	Polyamide fabric	Dyeing	*E. coli, S. aureus, P. aeruginosa, B. subtilis*	[45]
Hydrazono propanenitrile dyes	SSI, 120 °C, 20 MPa, 1–3 h With or without decalin cosolvent	UHMW polyethylene fiber	Dyeing	*E. coli, S. aureus, B. cereus*	[46]
Thymol	SSI, 35 °C, 10 and 20 MPa, 2–45 h	Cellulose acetate	5–72%	*S. aureus, C. albicans*	[20]
Thymol	SSI, 35 °C, 10 MPa, 2–32 h	Cellulose acetate	5–66%	*S. Typhimurium*, *S. Enteritidis*, *L. monocytogenes*, *L. ivanovii*, *L. innocua*, *Corynebacterium*, *R. equi*, *B. anthracis*, *B. cereus*, *B. subtilis*, *S. pneumoniae*, *S. pyogenes*, *S. aureus*, MRSA, *K. pneumoniae*, *P. aeruginosa*, *E. coli*, *Acinetobacter*, *P. mirabilis*	[47]
Carvacrol	SSI, 50 °C, 10–30 MPa, 2–18 h	Celulose acetate	5–60%	MRSA, *E.coli, Acinetobacter, B. anthracis, B. cereus, B. subtilis, Corynebacterium, K. pneumoniae, L. ivanovii, L. monocytogenes, R. equi, S. Enteritidis, S. pyogenes, S. pneumoniae*	[48]
Thymol	SSI, 35 °C, 15.5 MPa, 0.5–16 h	Cellulose acetate	8–64%	*S. aureus,* MRSA, *P. aeruginosa*	[49]
Carvacrol	SSI, 50 °C, 21 MPa, 0.5 and 2 h	Cellulose acetate	2.5–31.4%		[54]
Thymol	SSI, 40 °C, 10 MPa, 1 h	Cellulose nanofibril mats	4.1–8.3%	*E. coli, S. epidermidis, C. albicans*	[57]
Thymol	SSI, 35 °C, 10 MPa, 2–6 h	Chitosan-itaconic acid-methacrylic acid	1.0–4.6%		[58]
Thymol	SSI, 40 °C, 20 MPa, 3 h Near-critical, 30 °C, 10 MPa, 3 h	N-carboxybutylchitosan Agarose	0.8–2.5%		[59]
*d*-limonene	SSI, 40 °C, 20 MPa, 3 h	Poly(L-lactide-ran-cyclic carbonate)	0.15–5.3%		[60]
Roxithromycin	SSI, 40–70 °C, 8–30 MPa, 0.5–4 h	PLA	0.5–10.5%		[62]
Thymol	SSI, 40 °C, 9 and 12 MPa, 3 h	PLA	13.5–20.5%		[63]
Cinnamaldehyde	SSI, 40 °C, 9 and 12 MPa, 3 h	PLA	8–13%	*E. coli, S. aureus*	[64]
Thymol Cinnamaldehyde	SSI, 40 °C, 12 MPa, 3 h	PLA+nanoclay	17% 11%	*E. coli, S. aureus*	[65]
Thymol Thyme extract	SSI, 40 °C, 10 MPa, 1–15 h SFE-SSI, 40 °C, 10 MPa, 2–6 h	PLA/PCL	8–35.8% 4.3–5%	*E. coli, B. subtilis*	[66]
*Usnea lethariiformis* extract	SFE-SSI, 40 °C/30 MPa SFE; 35 °C/15 MPa SSI; 2 h flow + 1 h circ.	PCL	0.2–2.8%	MRSA, *L. innocua*	[28]
*Usnea lethariiformis* extract	SFE-SSI, 40 °C/30 MPa SFE; 35 °C/17 MPa SSI; 2 h flow + 1 h circ.	PCL+hydrohyapatite	1.7–5.9%	MRSA	[34]
Thymol	SSI and near-critical, 40 °C, 7–12 MPa, 4 h	LLDPE	1.5–3.8%		[67]
Eugenol	SSI, 40 °C, 10–15 MPa, 4 h	LLDPE	1–6%		[68]
Clove bud essential oil	SSI, 25–45 °C, 15 and 25 MPa, 4 h	LLDPE	1–4%		[69]
Thymol	SSI, 40 °C, 12 MPa, 1 h	LDPE+nanoclay	0.36–1.19%		[70]
Thymol	SSI, 40 °C, 9–12 MPa, 0.5–5 h	LDPE+nanoclay	0.82–1.62%	*S. aureus, E. coli*	[71]
Natamycin	SSI, 40 °C, 20 MPa, 2.5–14 h with or without ethanol cosolvent	Alginate	0.3–1.6%		[72]
Cinnamaldehyde	SSI, 35 °C, 15 and 2 MPa, 3 h	Starch	0.1–0.25%		[73]
Thymol	SSI, 35 °C, 15.5, 24 h	Starch	1.15–4.02%		[74]
Curry plant extract	SFE-SSI, 40 °C, 35 MPa, 5 h	Starch	1.26%		[33]
Lavandin essential oil	SSI, 40–50 °C, 10–12 MPa, 2 h	n-octenyl succinate modified starch	2.5–15%		[55]
Quaternary ammonium/N-chloramine polysiloxane	SSI, 50 °C, 28 MPa, overnight	PET	70 nm coating	*S. aureus, E. coli*	[75]
Quaternary ammonium compounds	SSI and chemical reaction, 100 °C, 41.4 MPa, 20 h Hexamethylene diisocyanate as a linker	Softwood		*E. coli*	[76]
Silver nitrate	HPAI, 20 °C, 12 MPa, 10 min SAI, 40 and 80 °C, 12 MPa, 10 min Ethanol solution of AgNO_3_	Polycarbonate	2.4 mg/kg 23.4 mg/kg	*E. coli*	[78]
Silver NPs (AgNO_3_ precursor)	SAI, 65 °C, 12 MPa, 3 h Ethanol solution, glucose as a reducer	Carbon nanomaterials		*E.coli*	[79]
Silver NPs (AgNO_3_ precursor)	SAI, 65 °C, 12 MPa, 3 h Ethanol solution, glucose as a reducer	Graphene oxide		*E. coli, S. aureus, L. anguillarum*	[80]
Ciprofloxacin loaded in IPN material	SSI or SAI + Polymerization SSI/SAI, 40 °C, 20–25 MPa, 20 min–16 h Polymerization, 75 °C, 30–36 MPa, 3 h	IPN material based on silicone elastomer and PHEMA	13–38% PHEMA	*S. aureus*	[82]
Ciprofloxacin loaded in IPN material	SSI + polymerization SSI, 40 °C, 20 MPa, 16 h	IPN material based on PDMS and PHEMA	25% PHEMA	*S. aureus*	[83]
	Polymerization, 75 °C, 30 MPa, 3 h				
Dicloxacillin Dicloxacillin and thioridazine	SSI + polymerization SSI, 40 °C, 20–25 MPa, 16 h Polymerization, 75 °C, 30 MPa, 3 h	IPN material based on silicone elastomer and PHEMA	25.29–41.68% PHEMA	*S. aureus,* MRSA	[84]
2-oxazoline-based oligomers	SSI + polymerization SSI, 40 °C, 18 MPa, 24 h Polymerization, 65 °C, 18 MPa, 20 h Reaction with tertiary amine, 40 °C, 18 MPa, 20 h	Chitosan		*E.coli, S. aureus*	[91]
Vancomycin	Foaming from solid dispersion	PCL and chitosan	1–5%	*E. coli, S. aureus*	[95]
	40 °C, 14 MPa, 1 h				
Thymol	SSI+ foaming in one step, 35 and 40 °C, 10–30 MPa, 2 h	PCL PCL+hydroxyapatite	12–18%		[94]
Thymol	SSI + foaming in one step, 25–50 °C, 7.5–15 MPa, 2–24 h	PLA PLGA	0.92–6.62%		[96]
Silver, gold and platinum NPs	Sc drying of metal-carrying gels Two steps: 5.3 MPa, 4 °C for 6 h, and 10 MPa, 40 °C for 0.5 h	Cellulose	Aerogel containing metal particles		[97]
Ca-Zn Ca-Zn-Ag	High-pressure gelation, 50 MPa, 24 h, room temperature; Sc drying of metal-carrying gel at 50 °C, 12 MPa, 2 h, 20 g/min CO_2_ flowrate	Calcium-alginate	Aerogel containing metal particles		[99]
TiO_2_ NPs	Sc drying of metal-carrying gel at 50–60 °C, 11–13 MPa, 5 h, 0.2 kg/h CO_2_ flowrate	Pectin	Aerogel containing NPs	*E. coli*	[100]
Cu_2_O and TiO_2_	Sc solvothermal process in ethanol as supercrit. fl., 243 °C, 6.4 MPa, 70 min		Cu_2_O-TiO_2_ nanocomposites	*A. baumannii*, *P. aeruginosa*, *E. coli*, *S. aureus*	[104]
Alkylthiols	Sc CO_2_ grafting at 100 °C and 10 MPa, 120 min	Oxide-free silicon	Deposited monolayer		[108]
TiO_2_ NPs	Physical treatment of fibers, 40 °C, 20 MPa, 60 min, fast decompression 0.80 MPa/min^−1^	Cotton fibers	NPs modified cotton		[109]
Isonicotinamide and copper(II) propanoate	Antisolvent precipitation (SAS)–ethanol soluton, 40 °C, 10 MPa, 1 mL/min		Ligand crystals produced		[110]
Gentamicin	Antisolvent precipitation from acetone solution at 10 MPa, 25 °C	GEN-AOT complex	Micronized solid	*E. coli*	[111]
Caffeic acid phenethyl ester	RESOLV–ethanol solution at 17.3 MPa and 50 °C; nozzle at 80 °C		NPs produced	*P. aeruginosa, C. albicans, L. monocytogenes*	[112]
Lavandin essential oil	PGSS drying, 104–130 °C, 6–10 MPa PGSS, 70 °C, 6–8.5 MPa	Soybean lecithin, n-octenyl succinic anhydride modified starch, PCL	Oil encapsulated in polymer	*E. coli, S. aureus, B. Baccereus*	[113]
Lavandin essential oil	PGSS, 76–84 °C, 5.4–8.5 MPa PGSS drying, 108–127 °C, 9–12.4 MPa	PEG n-octenyl succinic anhydride modified starch	Oil encapsulated in polymer		[114]
Vancomycin	SuperLip, 40 °C, 10 MPa	Phospholipids	Liposomes		[116]
Amoxicillin	SuperLip, 40 °C, 10 MPa	Phospholipids	Liposomes	*E. coli*	[117]
Ampicillin Ofloxacin	SuperLip, 40 °C, 10 MPa	Phospholipids	Liposomes		[118]
Amoxicillin	SuperLip, 40 °C, 10 MPa Sc Drying, 35 °C, 20 MPa, 6 h, 1 kg/h scCO_2_ flowrate	Phospholipids Alginate	Liposomes entrapped in aerogel		[119]

**Table 2:** Cited references related to the techniques applied.

High-Pressure Methodology	Reference
Supercritical Solvent Impregnation (SSI)	[20,22,23,25,26,27,28,29,30,31,32,33,34,36,37,38,39,40,44,45,46,47,48,49,54,55,57,58,59,62,63,64,65,66,67,68,69,70,71,72,73,74,75,94,96]
Supercritical Assisted Impregnation (SAI) High-pressure Assisted Impregnation (HPAI)	[78,79,80] [78]
SSI/SAI + polymerization	[82,83,84,91]
SSI/SAI + chemical reaction other than polymerization	[76,91,108]
Supercritical foaming	[28,34,94,95,96]
Supercritical drying	[97,99,100,119]
Supercritical solvothermal process ^1^	[104]
Antisolvent techniques	[110,111]
RESOLV	[112]
PGSS	[113,114]
Physical surface modification	[109]
Liposome formation (SuperLip)	[116,117,118,119]

^1^ Supercritical ethanol.
